# Potential drug targets for tumors identified through Mendelian randomization analysis

**DOI:** 10.1038/s41598-024-62178-w

**Published:** 2024-05-18

**Authors:** Na Song, Pingyu Shi, Kai Cui, Liqun Zeng, Ziwei Wang, Wenyu Di, Jinsong Li, Yanwu Fan, Zhanjun Li, Jinghang Zhang, Wei Su, Haijun Wang

**Affiliations:** 1 Department of Pathology, Xinxiang Key Laboratory of Precision Medicine, The First Affiliated Hospital of Xinxiang Medical University, Jiankang Road No.88, Xinxiang, 453100 China; 2 Department of Pathology, School of Basic Medical Sciences, Xinxiang Medical University, Jinsui Road No. 601, Xinxiang, 453000 China; 3 State Key Laboratory for Diagnosis and Treatment of Severe Zoonotic Infectious Diseases, Key Laboratory for Zoonosis Research of the Ministry of Education, Institute of Zoonosis, and College of Veterinary Medicine, Jilin University, Changchun, 130062 China

**Keywords:** Drug target, Biomarker, Plasma protein, Tumor, Mendelian randomization, Cancer, Drug discovery

## Abstract

According to the latest cancer research data, there are a significant number of new cancer cases and a substantial mortality rate each year. Although a substantial number of clinical patients are treated with existing cancer drugs each year, the efficacy is unsatisfactory. The incidence is still high and the effectiveness of most cancer drugs remains unsatisfactory. Therefore, we evaluated the human proteins for their causal relationship to for cancer risk and therefore also their potential as drug targets. We used summary tumors data from the FinnGen and cis protein quantitative trait loci (cis-pQTL) data from a genome-wide association study, and employed Mendelian randomization (MR) to explore the association between potential drug targets and nine tumors, including breast, colorectal, lung, liver, bladder, prostate, kidney, head and neck, pancreatic caners. Furthermore, we conducted MR analysis on external cohort. Moreover, Bidirectional MR, Steiger filtering, and colocalization were employed to validate the main results. The DrugBank database was used to discover potential drugs of tumors. Under the threshold of False discovery rate (FDR) < 0.05, results showed that S100A16 was protective protein and S100A14 was risk protein for human epidermal growth factor receptor 2-positive (HER-positive) breast cancer, phosphodiesterase 5A (PDE5A) was risk protein for colorectal cancer, and melanoma inhibitory activity (MIA) was protective protein for non-small cell lung carcinoma (NSCLC). And there was no reverse causal association between them. Colocalization analysis showed that S100A14 (PP.H4.abf = 0.920) and S100A16 (PP.H4.abf = 0.932) shared causal variation with HER-positive breast cancer, and PDE5A (PP.H4.abf = 0.857) shared causal variation with colorectal cancer (CRC). The MR results of all pQTL of PDE5A and MIA were consistent with main results. In addition, the MR results of MIA and external outcome cohort were consistent with main results. In this study, genetic predictions indicate that circulating S100 calcium binding protein A14 (S100A14) and S100 calcium binding protein A16 (S100A16) are associated with increase and decrease in the risk of HER-positive breast cancer, respectively. Circulating PDE5A is associated with increased risk of CRC, while circulating MIA is associated with decreased risk of NSCLC. These findings suggest that four proteins may serve as biomarkers for cancer prevention and as potential drug targets that could be expected for approval.

According to the latest cancer survey data, there are a significant number of new cancer cases and substantial mortality rates each year. Malignant tumors such as lung cancer, colorectal cancer, and pancreatic cancer rank in the top ten in both incidence and mortality rates for both male and female^1^. Furthermore, prostate cancer remains the leading cause of death among men. Despite a significant decline in prostate cancer mortality rates, it still stands as the second leading cause of cancer-related deaths^2^. Similarly, although survival rates for breast cancer have improved significantly over the past few decades, it remains the most common cancer and the second deadliest cancer among female^3^. Therefore, cancer prevention and treatment are important in the field of scientific research. However, the identification of biomarkers for cancer prevention is still insufficient, and the unique mechanisms within the tumor microenvironment pose challenges for drug treatments^4–6^. Currently, commonly used anti-cancer drugs include 5-fluorouracil, capecitabine, paclitaxel, gemcitabine, and others. However, the effectiveness of these drugs remains limited to a small subset of patients^7,8^. In recent years, studies have attempted to discover novel and efficient cancer therapeutic drugs^9,10^. However, some findings encountered obstacles in the process of translating into clinical outcomes. For example, some anti-cancer drugs may encounter issues such as insufficient efficacy, ineffectiveness, or adverse reactions^11^. Revealing precancerous markers and identifying potential therapeutic targets may offer new insights into the future prevention and treatment of cancer.

Proteins play crucial roles in various biological processes in the human body, as well as in the progression of cancer^12,13^. Currently, due to the vital functions of proteins, multiple drug targets are primarily concentrated on proteins. In recent years, MR studies have been widely employed in drug target screening and drug repurposing^14,15^. MR is a recently emerging research method that employs genetic instrumental variables for causal association analysis^16,17^. Single nucleotide polymorphisms (SNPs) identified through genome-wide association studies are used as instrumental variables to assess the causal association between exposure and outcome. Here, the study of human plasma protein data using MR may help to uncover underlying genetic factors in tumors.

In our study, we first performed a two-sample MR analysis with plasma protein as exposure and 9 tumors in the FinnGen database as outcomes. Our results were then further explored by external queue validation, reverse MR, and co-localization analysis. Finally, four plasma proteins are being considered as potential drug targets for tumors, and Drugs that identify potential targets based on DrugBank database are considered potential therapeutics for tumors.

## Materials and methods

### Mendelian randomization (MR)

**Figure 1:**
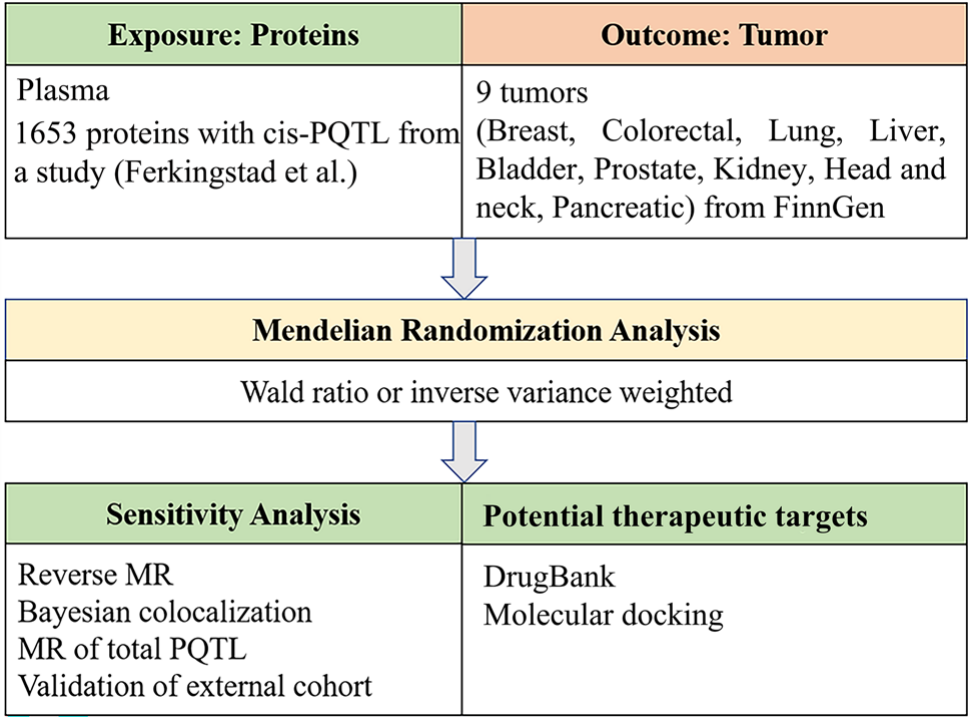
Study design for identification of plasma proteins causally associated with tumors. pQTL: protein quantitative trait loci.

Mendelian Randomization (MR) is a novel analytical method that has emerged in recent years, utilizing statistical techniques to assess the impact of a specific factor on human diseases^18^. It utilizes instrumental variable to analysis, aiming to assess causal relationships between exposures and outcomes using non-experimental data. In MR analysis, genetic variation SNPs are considered instrumental variables. When DNA is passed from parents to offspring, alleles segregate independently. This method is akin to random assignment in randomized controlled trials (RCTs), with the purpose of simulating RCTs to minimize the risk of confounding^19^. However, before conducting MR analysis, three assumptions need to be met. First, the instrumental variables must be strongly correlated with the exposure factor. Second, the instrumental variables must be independent of confounding factors. Third, the instrumental variables should only affect the outcomes through the exposure factor^20^. In accordance with the principles and assumptions of Mendelian randomization, we have crafted a flowchart outlining the analysis procedure for this study (Fig. 1).

### pQTL and GWAS data

The study design was shown in Fig. 1. We conducted a MR analysis to explore the causal association between plasma protein concentration (n = 4917) and nine types of tumors, including breast, colorectal, lung, liver, bladder, prostate, kidney, head and neck, pancreatic caners. Summary-level data of plasma pQTL was obtained from a comprehensive genome-wide association study (GWAS) published by Ferkingstad et al.^21^. In addition, we performed the analysis with cis-pQTLs as they are known for their resistance to horizontal pleiotropy^22^. Previously, a study has identified several potential drug targets for acute pancreatitis through MR analysis of cis-pQTLs for 4719 plasma proteins^23^. Here, we employed the same plasma protein screening approach as follows: Firstly, a rigorous association screening was performed on 4917 plasma protein pQTL (*p* < 5e-8), which be advantageous for excluding weak instrumental variables. Secondly, SNPs with linkage disequilibrium (LD) were eliminated based on the criteria of *r*^2^ = 0.001. Thirdly, to ensure that SNPs are not pleiotropy, the retained SNPs are limited to those with cis (Gene locus ± 1Mb). In addition, due to algorithmic reasons, SNPs with a low minor allele frequency (MAF) exhibit higher error rates, hence it is necessary to set the MAF threshold to 0.01. Additionally, we utilized PhenoScanner, a database containing published literature on GWAS data information, to control for confounding factors^24^. F-value is an indicator of the correlation between an instrumental variable and exposure, and the SNPs (F-value > 10) are strongly correlated with exposure^25^.

The tumor GWAS data were obtained from the R9 version of the FinnGen database (https://www.finngen.fi/en/access_results) (Supplement Table S1)^26^, including malignant neoplasm of breast (HER-negative) (num_cases = 5965, num_controls = 167,017), malignant neoplasm of breast (HER-positive) (num_cases = 9698, num_controls = 167,017), colorectal cancer (num_cases = 6509, num_controls = 287,137), non-small cell lung cancer (num_cases = 4901, num_controls = 287,137), small cell lung cancer (num_cases = 676, num_controls = 167,017), hepatocellular carcinoma (num_cases = 453, num_controls = 287,137), clear cell adenocarcinoma of kidney (num_cases = 901, num_controls = 167,017), malignant neoplasm of bladder (num_cases = 2053, num_controls = 167,017), malignant neoplasm of head and neck (num_cases = 2131, num_controls = 287,137), adenocarcinoma and ductal carcinoma of pancreas (num_cases = 692, num_controls = 287,137), malignant neoplasm of prostate (num_cases = 13,216, num_controls = 119,948). All data were derived from public databases and approved by the ethics review committee.

### Data for validation

The GWAS summary data for colorectal cancer and lung cancer were derived from the UK Biobank database and were obtained from the Open GWAS online database (https://gwas.mrcieu.ac.uk). The study included a total of 5,657 cases and 372,016 controls for colorectal cancer, as well as 2,671 cases and 372,016 controls for lung cancer. Moreover, the GWAS summary data of breast cancer were obtained from the Breast Cancer Association Consortium (BCAC)^27^, encompassing a total of 69,501 cases and 105,974 controls. These data were used to validate the primary results.

### MR analysis

We conducted a two-sample MR analysis using the retained SNPs to identify potential therapeutic targets for tumors. In this study, plasma protein concentration was employed as the exposure factor, while tumor served as the outcome factor. The methods of Inverse-variance weighted (IVW) and the Wald ratio were employed to assess the causal association between plasma protein concentration and tumors^28^. The Wald ratio was employed for the analysis of a single SNP, while the IVW method was utilized to evaluate multiple SNPs. The main results were screened based on the threshold condition of false discovery rate (FDR) < 0.05^29^, and the Benjamini–Hochberg method was employed for FDR calculation. In addition, we utilized the phenoscanner platform (http://www.phenoscanner.medschl.cam.ac.uk/) to fulfill the assumptions of independence and exclusivity in MR analysis^30^. SNPs associated with tumor risk factors and those directly associated with tumors were eliminated. Afterwards, we utilized the ‘harmonise_data’ and ‘mr’ function in TwoSampleMR to extract the same SNPs in the outcome and obtained the results. And the accuracy of the direction was verified by steiger test. Furthermore, we validated the main results with additional tumor GWAS data. For external queue validation results, the threshold was adjusted to *p* < 0.05. Finally, MR Analysis of all pQTL data (cis-pQTL and trans-pQTL) was also used as a sensitivity analysis in order to verify the main results. The all methods used for the sensitivity analysis are shown in Fig. 1. Additionally, to verify the reliability of the results, we conducted a statistical power analysis for the main outcomes using the online website https://sb452.shinyapps.io/power.

### Reverse MR analysis

In order to exclude the bias caused by reverse causality, we conducted a bidirectional MR analysis. According to the same screening conditions (*p* < 5e-8, kb = 10,000, *r*^2^ = 0.001), SNPs were extracted from the GWAS data of breast tumor, colorectal cancer, and non-small cell lung cancer in the FinnGen database, with tumor as exposure and plasma protein concentration as outcome. IVW, MR-Egger, weighted median, simple mode, and weighted mode were used as the primary analysis methods^31–33^. *p* < 0.05 was considered statistically significant.

### Colocalization analysis

Colocalization analysis is a method of investigating whether a causal variation is shared between two traits and is used to enhance the results of MR Analysis^34^. The Bayesian colocalization method provides posterior probabilities of five hypotheses^35^. To assess the presence of shared causal variation in a specific genomic region between the two phenotypes, Bayesian colocalization analysis (pQTL-GWAS) was performed using SNPs located within 500 Mb from the lead SNP of all cis-pQTL with MAF > 0.01^23^. There are four hypotheses for co-localization analysis. H0: Phenotype 1 and Phenotype 2 are not significantly associated with any SNP loci in a specific genomic region. H1/H2: Phenotype 1 or Phenotype 2 is significantly associated with SNP loci in a specific genomic region. H3: Both Phenotype 1 and Phenotype 2 are significantly associated with SNP loci in a specific genomic region, but driven by different causal variant positions. H4: Both Phenotype 1 and Phenotype 2 are significantly associated with SNP loci in a specific genomic region, and driven by the same causal variant position. If H4 has a higher probability in statistical terms, it suggests that the significant signal locus can influence the phenotype. In this study, based on the coloc package, we evaluated the posterior probability of hypothesis 4 (PP.H4). PP.H4 > 0.8 was identified as being associated with a specific genomic region through shared causal variation between the protein and the tumor^36^, which is strong evidence for co-localization. 0.4 < PP.H4 < 0.8 was regarded as moderate co-localization.

### Potential target drugs

Plasma proteins with strong co-localization and favorable results in sensitivity analyses were selected as first-class targets. In addition, the STRING database (https://string-db.org/) and DrugBank (https://go.drugbank.com/) were utilized to investigate whether discovered cancer therapeutic drug targets interact with the first-class targets^37^. furthermore, the DrugBank database is being used separately to explore potential cancer therapy drugs for first-class targets. The molecular docking of potential target drugs was performed on MCULE online platform with default settings (https://mcule.com/apps/1-click-docking/)^38–41^. The platform will generate top four ranked of molecular docking results according to the drug molecules and targets provided, and we selected Rank 1 to prepare the figures.

## Results

### Proteomic screening of tumor-causing proteins

**Figure 2:**
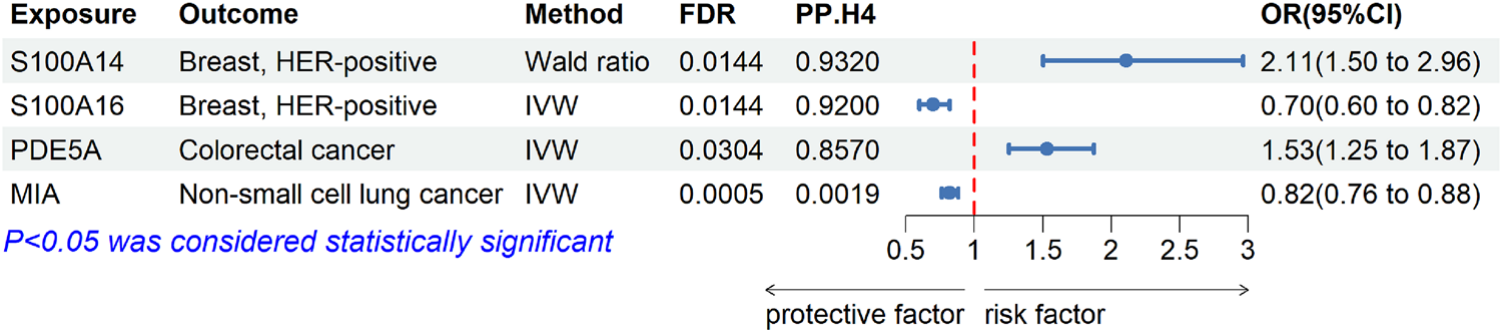
MR results for plasma proteins on the risk of tumors. Causal association of four plasma proteins (S100A14, S100A16, PDE5A, MIA) with tumors based on MR analysis with Wald ratio and Inverse Variance Weighted methods.

In our results, 5365 cis-pQTL of a total of 1743 proteins were used for follow-up analysis after a series of screening (Supplement Table S2). However, after extracting SNPs in the outcome, some lacked SNPs, leaving SNPs with only 1653 plasma proteins in every tumor. A total of 1005 proteins were causally associated with tumors at the *p* < 0.05 threshold (Supplement Table S3), while there were 6 significant outcomes at the FDR < 0.05 threshold (NCAN, GREM1, S100A16, S100A14, PDE5A, MIA), and only the following 4 proteins (S100A16, S100A14, PDE5A, MIA) were causally associated with tumors after removing the SNPs associated with the outcomes by phenoscanner (Fig. 2). An instrumental variable of NCAN, rs2228603, was associated with nonalcoholic fatty liver disease. The instrumental variables of GREM1, rs4779584 and rs144674978, were associated with colorectal cancer. The statistical significance of NCAN and GREM1 did not reach the threshold of FDR < 0.05. In non-small cell lung cancer, rs2279011 was excluded because of its association with smoking. There were causal associations between S100A14 (Wald ratio, OR = 2.11, 95% CI, 1.50–2.96, FDR = 0.0144) and S100A16 (IVW, OR = 0.70, 95% CI, 0.60–0.82, FDR = 0.0144) and HER-positive breast tumors, between PDE5A (IVW, OR = 1.53, 95% CI, 1.25–1.87, FDR = 0.0304) and colorectal cancer, and between MIA (IVW, OR = 0.82, 95% CI, 0.76–0.88, FDR = 0.0005) and non-small cell lung cancer. And they had the correct causal direction (*p* < 0.05). There was no heterogeneity (SNP > 2) and pleiotropy (SNP > 3) in results (Supplement Table S3). These results suggest that S100A16 may be a risk protein for HER-positive breast cancer, S100A14 may be a protective protein for HER-positive breast cancer, PDE5A may be a risk protein for colorectal cancer, and MIA may be a risk protein for non-small cell lung cancer. Additionally, the results have been validated as reliable through statistical power analysis (Supplement Table S4).

### Sensitivity analysis of tumor causal proteins

**Figure 3:**
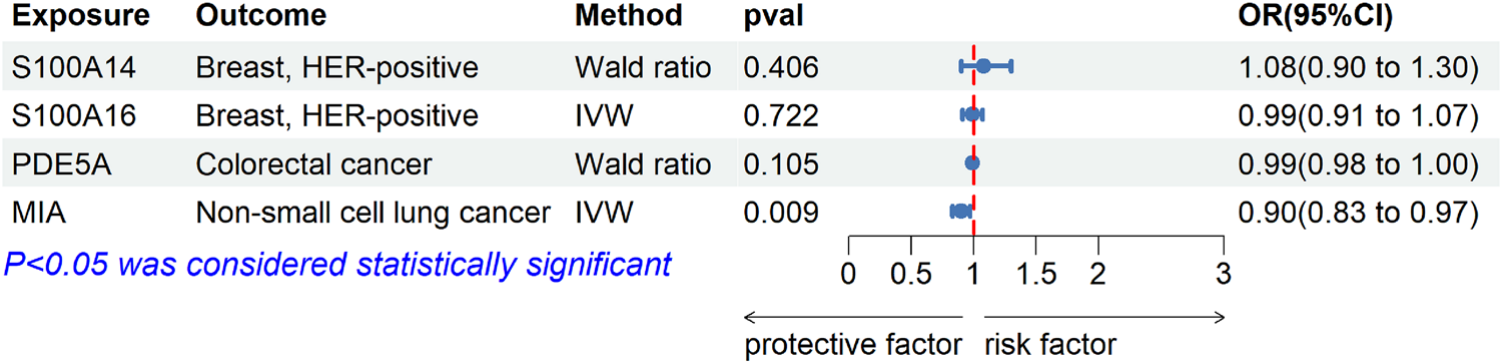
External queue validation of MR results for plasma proteins on the risk of tumors. External data for the four plasma proteins came from the UK Biobank and the Breast Cancer Association Consortium (BCAC).

**Figure 4:**
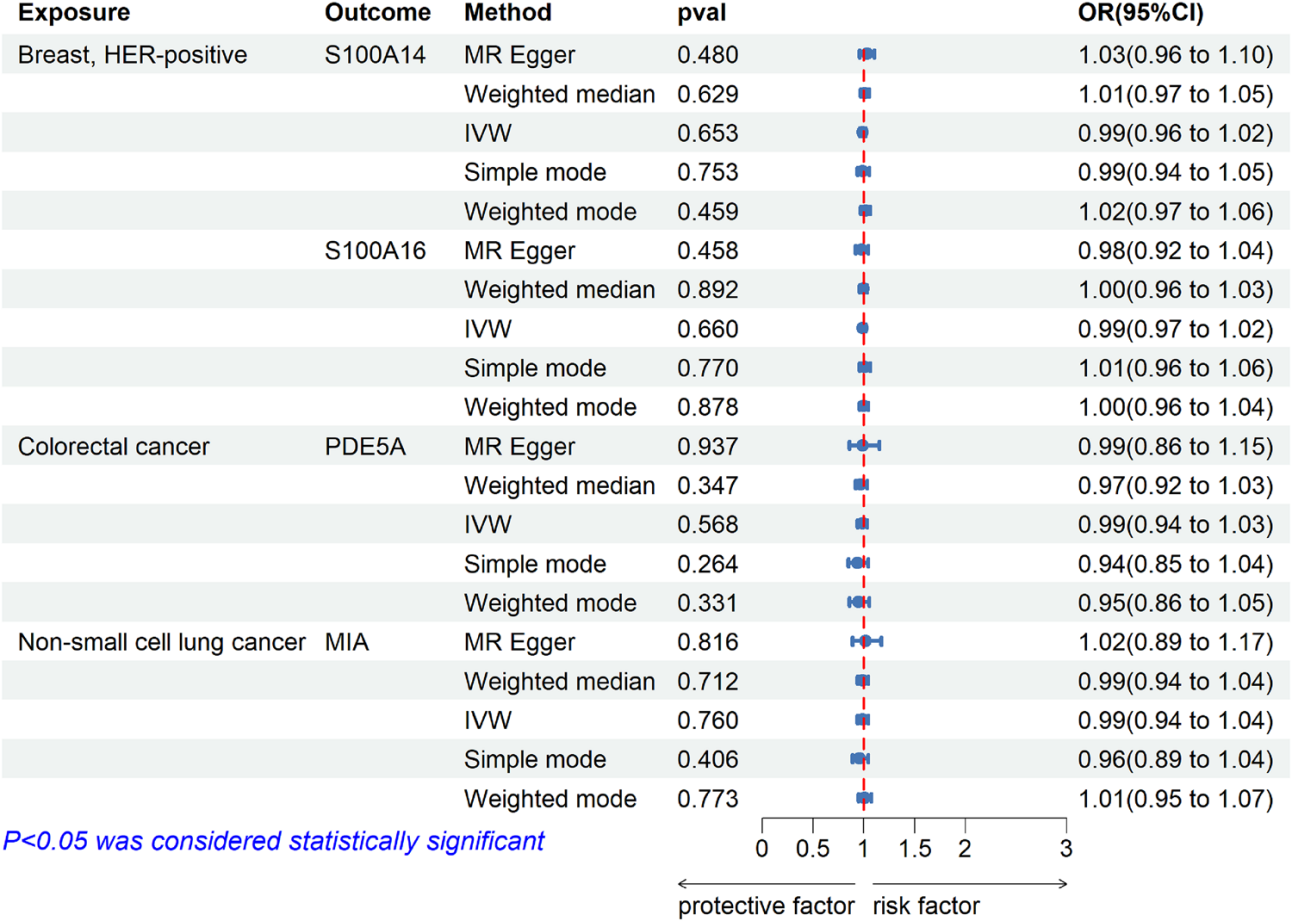
Reverse MR results for plasma proteins on the risk of tumors. There was no reverse causal effect on the primary MR results.

**Figure 5:**
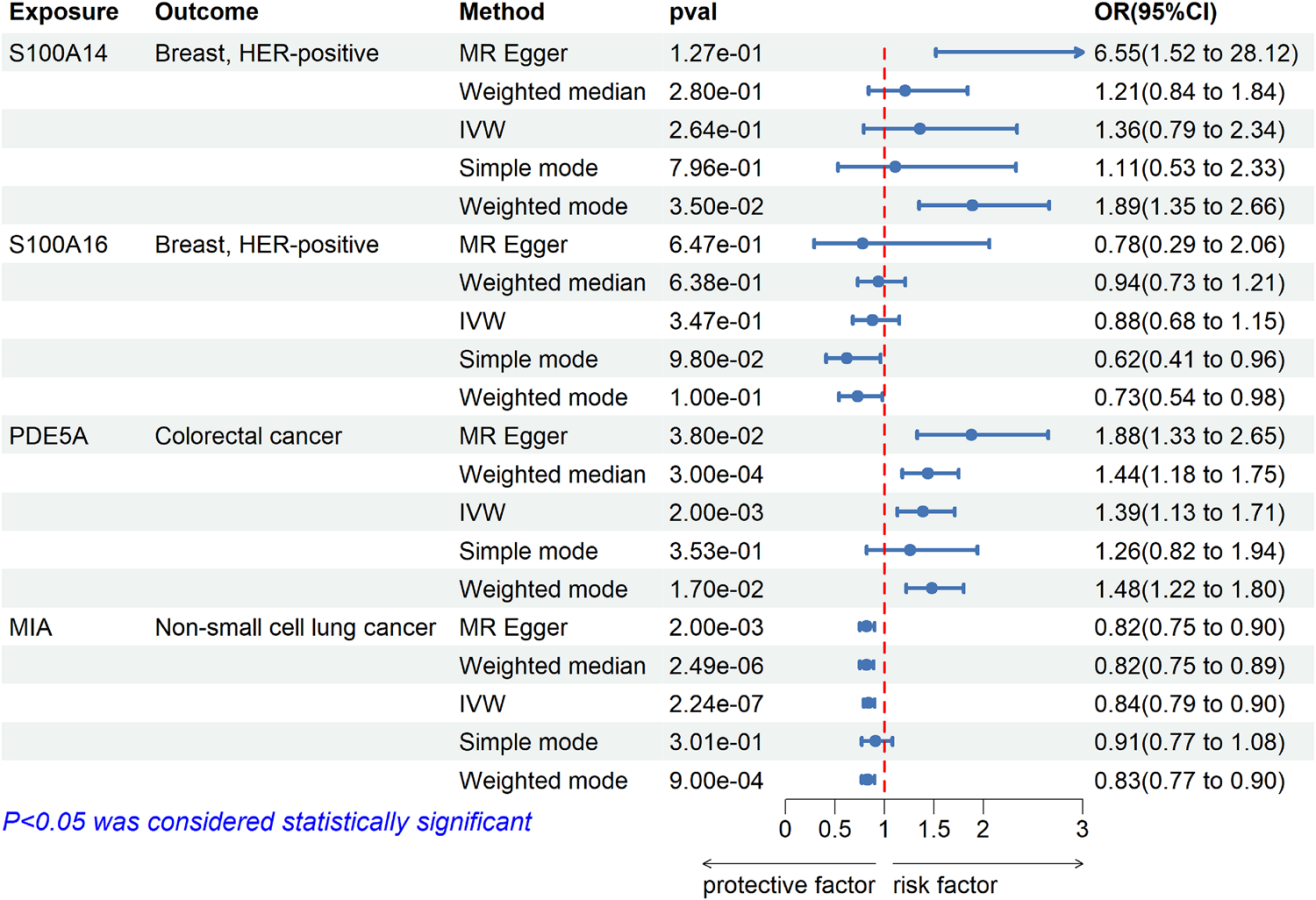
MR results of all pQTL for plasma proteins on the risk of tumors. The results of PDE5A and MIA were replicated in all PQTL (cis-pQTL and Trans-pQTL) data as exposed.

In our findings, four proteins were ultimately identified as potential drug targets for tumors, including S100A16, S100A14, PDE5A, and MIA. In addition, Reverse validation, Bayesian colocalization, MR analysis of the total pQTL, and validation of external queue were used as sensitivity analyses. In external queue validation, the results for S100A16 (IVW, OR = 0.99, 95% CI, 0.91–1.07, *p* = 0.722), S100A14 (Wald ratio, OR = 1.08, 95% CI, 0.90–1.30, *p* = 0.406) and PDE5A (Wald ratio, OR = 0.99, 95% CI, 0.98–1.00, *p* = 0.105) were not replicated, while the results for MIA (IVW, OR = 0.90, 95% CI, 0.83–0.97, *p* = 0.009) were replicated in non-small cell lung cancer (Fig. 3). And there was no heterogeneity and pleiotropy (*p* > 0.05) (Supplement Table S3). Moreover, in reverse MR Analysis, tumors were considered exposure, and plasma proteins were considered outcome. No causal association was found between tumors and plasma protein (Fig. 4). Meanwhile, to validate our main results, all pQTL data of four plasma proteins (cis-pQTL and trans-pQTL) were used for MR Analysis, the same findings were observed for plasma proteins PDE5A (IVW, OR = 1.39, 95% CI, 1.13–1.71, *p* = 2e-3) and MIA (IVW, OR = 0.84, 95% CI, 0.79–0.90, *p* = 2e-7) (Fig. 5). Interestingly, the results of colocalization analysis showed that S100A16 (coloc, PP.H4.abf = 0.933), S100A14 (coloc, PP.H4.abf = 0.913), and PDE5A (coloc, PP.H4.abf = 0.855) had the shared causal variation with HER-positive breast and colorectal cancers, respectively (Fig. 2). These results further validated the causal association between four plasma proteins and tumors.

### Potential therapeutic drugs for tumors

**Figure 6:**
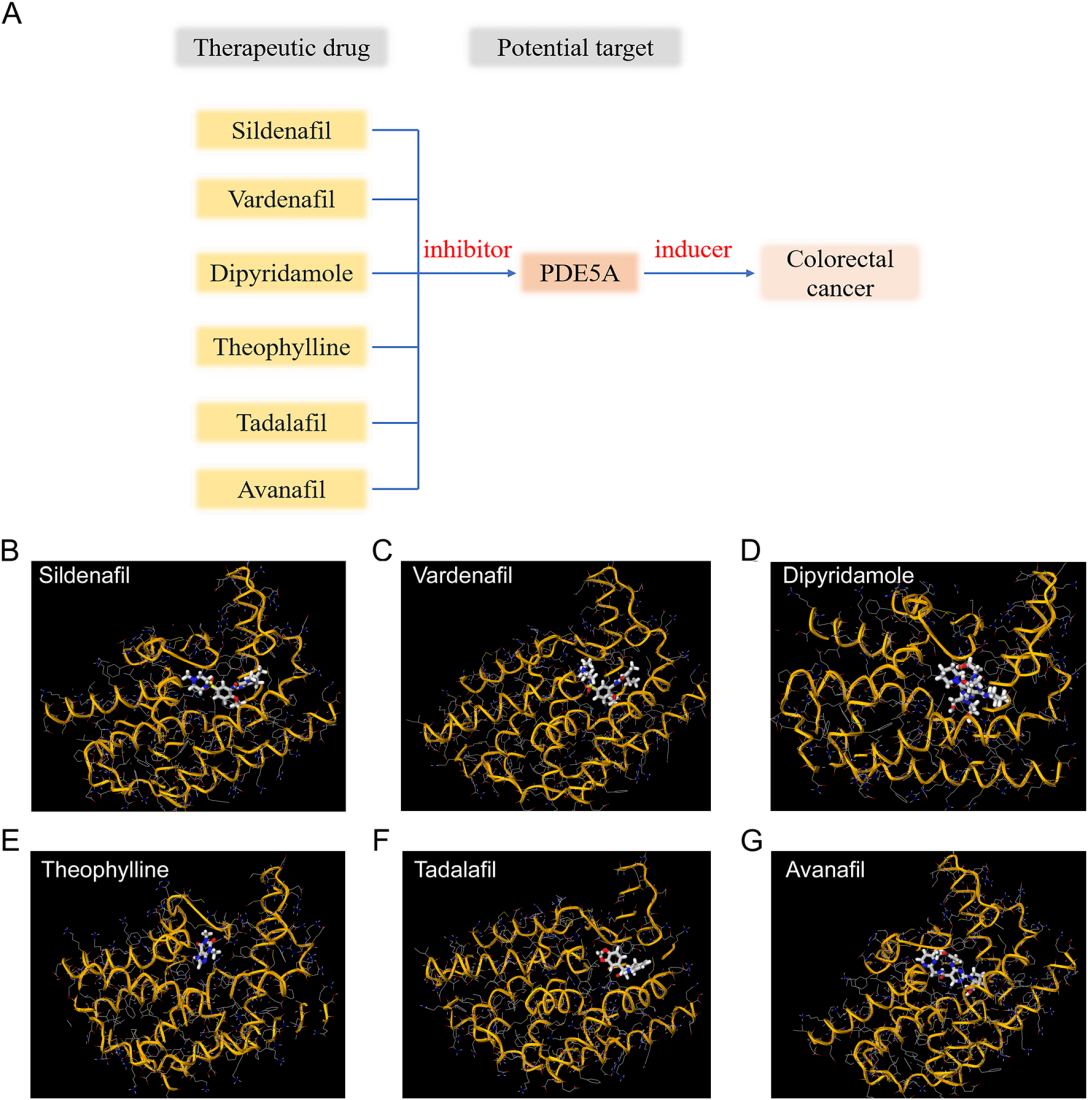
Potential target drugs for colorectal cancer based on MR analysis. Potential target drugs came from DrugBank database. (**A**) Potential drugs for the target PDE5A in colorectal cancer; (**B-G**) Molecular docking results of Sildenafil, Vardenafil, Dipyridamole, Theophylline, Tadalafil and Avanafil based on MCULE online platform.

Based on the above results, we considered PDE5A as a first-class therapeutic target, S100A16, S100A14 and MIA as second-class targets, and the other proteins with *p* < 0.05 threshold as third-class targets. While indirect potential drug targets were not identified through STRING, we discovered drugs in the medication database that may interact with the targets. Through the search of DrugBank database, we found that target of the marketed drugs Sildenafil, Vardenafil, Dipyridamole, Theophylline, Tadalafil and Avanafil was PDE5A, and they were used as inhibitors of PDE5A (Fig. 6). These results suggest that PDE5A inhibitor drugs may have a preventive effect as well as a potential therapeutic effect on colorectal cancer. However, there were no suitable drugs targeting S100A16, S100A14 and MIA. These findings suggest that targeting PDE5A holds significant potential as a therapeutic target for colorectal cancer in future clinical investigations.

## Discussion

In this study, we primarily used the two-sample MR method to investigate the causal association between human plasma protein concentration and tumors, aiming to discover potential drug therapeutic targets and cancer prevention biomarkers. Based on our research, we set a criterion: Proteins with strong colocalization and robust sensitivity were identified as potential first-class targets, while proteins that met only one condition were identified as second-class potential targets. Ultimately, based on our analysis, S100A16 and S100A14 were validated as potential second-class drug targets for HER-positive breast cancer, PDE5A as potential first-class drug targets for colorectal cancer, and MIA as potential second-class drug targets for non-small cell lung cancer. In these findings, the causal relationship between MIA and lung cancer was further confirmed in external queue (ieu-b-4954), providing additional credibility to the potential drug targets. Our analysis also revealed that MR Analyses of trans-pQTL and cis-pQTL for PDE5A and MIA were consistent with the main results.

Firstly, in order to eliminate the effects of confounding factors and horizontal pleiotropy, PhenoScanner was used to remove SNPs associated with outcomes and confounding factors before main analysis. Secondly, evaluation results based on data from previous drug development programs showed that target indication pairings identified by MR and co-localization were more likely to be approved^42^. To assess the potential of these findings as targets for tumor drug therapy, we further evaluated the causal association between plasma proteins and tumors through colocalization analysis^43^. HH.P4 > 0.8 was considered evidence of strong colocalization. Three proteins identified by MR, S100A16, S100A14 and PDE5A, might share causal variables with HER-positive breast cancer and colorectal cancer. In addition, Reverse causation MR analysis was performed to remove the effects of reverse causation. Results showed no reverse causal association between plasma proteins and tumors.

In our analysis, S10014, S100A16 and MIA were regarded as second-class target of HER-positive breast cancer. S100A14 and S100A16 both belong to calcium-binding protein. These proteins were involved in various biological functions of the human body, such as cell signal transduction, cell proliferation, and apoptosis^44,45^. Studies had shown that S100A14 might be related to the breast cancer metastasis by promoting expression of chemokines CCL2 and CXCL5^46^. Mechanistically, S100A14 activated NF-κB signaling to upregulate chemokine expression. This was consistent with our findings that S100A14 was considered a risk protein for HER-positive breast cancer. Similarly, another study showed that S100A14 was an independent prognostic factor in triple-negative breast cancer^47^. In addition, expression of S100A16 activated epithelial-mesenchymal transition and promoted breast cancer progression^48^, which was contrary to our findings. Interestingly, a study had shown that co-expression of S100A16 and S100A14 in breast cancer promoted the invasive ability of cancer cells; overexpression and knockdown of S100A14 could affect the expression of S100A16^49^. Therefore, we speculated that S100A14 might play a leading role in breast cancer. In addition, MIA was regarded as second-class target of non-small cell lung cancer in our analysis. MIA was a small secreted protein that was primarily secreted by but not limited to melanoma^50^. Overexpression of MIA not only promoted melanoma metastasis but also activated the invasive ability of pancreatic cancer cells. However, the association between MIA and lung cancer had not been studied so far. Moreover, PDE5A were regarded as first-class target of colorectal cancer. So far, multiple studies had shown PDE5A as a potential therapeutic target for tumors. PDE5A was overexpressed in breast cancer stroma and affected the growth of breast cancer cells by inducing the expression of chemokine CXCL16^51^. In addition, the use of the PDE5A inhibitors sildenafil or vardenafil enhanced apoptosis of human castration-resistant prostate cancer cells^52^. In colorectal cancer, the use of PDE5A inhibitors had been found to be associated with a favorable prognosis and lower metastasis rate in male with colorectal cancer^53^. To our knowledge, no previous studies had used MR combined with colocalization to explore the causal association between PDE5A and colorectal cancer. Our results provide evidence for the clinical translation of PDE5 inhibitors in treatment of colorectal cancer.

Although the number of potential therapeutic targets identified had increased in recent years, that had not translated into clinical outcomes. Based on the phenomenon, we used cis-pQTL for MR and colocalization analysis to identify potential therapeutic targets for tumors, which will be more likely to be approved.

Despite our study identifying potential targets and drugs advantageous for clinical cancer treatment, however, there are some limitations in this analysis. The limitations of this study are as follows: First, we extracted 4917 protein pQTLs from Ferkingstad et al.^21^ for exposure in MR analysis. After preliminary processing, only 1653 plasma proteins remained for analysis, and many potential plasma proteins still await validation. In addition, external data of exposure were not further verified. The results may be subject to some bias. Second, cis-pQTL was reserved for analysis, and some pQTL had only one instrumental variable, so it could not be analyzed for heterogeneity or pleiotropy. Although we subsequently added trans-pQTL, there may be some bias in the results. However, we subsequently enhanced the accuracy of the results by employing colocalization analysis. Thirdly, our study data originated from the decode data and the FinnGen cohort, both of which consist of European populations. Therefore, our results may be applicable primarily to European populations. In addition, it is well known that different tumor subtypes have different therapeutic strategies based on different information such as tumor marker expression, driver gene mutation, and tumor stage and grade, etc. In this study, because FinnGen database did not provide further clinical classification based on clinicopathological information such as gene alteration and tumor stages/grade, etc., we could not analyze the correlation between clinical subtypes of related tumors. Fourth, as we focused on exploring potential drug targets for cancer, the conclusions drawn have not been validated through clinical trials. Therefore, these results require further investigation.

## Conclusions

Overall, our study employed MR analysis to investigate causal relationships between plasma proteins and nine types of cancer. We found that elevated S100A14 in circulation increased the risk of HER-positive breast cancer, while S100A16 decreased the risk of HER-positive breast cancer. PDE5A was associated with an increased risk of CRC, and MIA was linked to a reduced risk of SCLC. Additionally, we identified PDE5A, a target for erectile dysfunction drugs, as a potential therapeutic target for CRC, suggesting that such medications may serve as novel treatments by inhibiting PDE5A. However, further research is needed to validate these findings and understand their specific roles in tumor diagnosis and drug target development.

### Supplementary Information

Supplementary Information 1.

Supplementary Information 2.

Supplementary Information 3.

Supplementary Information 4.

## Data Availability

All data for this study is available from the corresponding author upon reasonable request.
